# Simultaneous and Direct Determination of Vancomycin and Cephalexin in Human Plasma by Using HPLC-DAD Coupled with Second-Order Calibration Algorithms

**DOI:** 10.1155/2012/256963

**Published:** 2012-04-22

**Authors:** Le-Qian Hu, Chun-Ling Yin, Ya-Hui Du, Zhi-Peng Zeng

**Affiliations:** School of Chemistry and Chemical Engineering, Henan University of Technology, Zhengzhou 450001, China

## Abstract

A simple, rapid, and sensitive method for the simultaneous determination of vancomycin and cephalexin in human plasma was developed by using HPLC-DAD with second-order calibration algorithms. Instead of a completely chromatographic separation, mathematical separation was performed by using two trilinear decomposition algorithms, that is, PARAFAC-alternative least squares (PARAFAC-ALSs) and self-weight-alternative-trilinear-decomposition- (SWATLD-) coupled high-performance liquid chromatography with DAD detection. The average recoveries attained from PARAFAC-ALS and SWATLD with the factor number of 4 (*N* = 4) were 101 ± 5% and 102 ± 4% for vancomycin, and 96 ± 3% and 97 ± 3% for cephalexininde in real human samples, respectively. The statistical comparison between PARAFAC-ALS and SWATLD is demonstrated to be similar. The results indicated that the combination of HPLC-DAD detection with second-order calibration algorithms is a powerful tool to quantify the analytes of interest from overlapped chromatographic profiles for complex analysis of drugs in plasma.

## 1. Introduction

 Vancomycin belongs to a group of antibiotics called glycopeptides. It is used for severe staphylococcal and streptococcal infections in patients intolerant of *β*-lactam antibiotics, in methicillin-resistant staphylococcal infections, and against gram-positive organisms with multiple resistances to antibiotics [1]. Vancomycin has also been reported to cause concentration-dependent renal toxicities, and therefore monitoring of plasma vancomycin concentration is essential to obtain sufficient efficacy and to prevent toxic side effects [2]. On the other hand, as a narrow spectrum antimicrobial, vancomycin has led to considerable interest in exploring antimicrobial combination therapy. The presence of significant in vitro synergy (time-kill method) between levofloxacin and vancomycin against clinical isolates of penicillin resistant pneumoniae was encouraging [3]. This case demonstrates that high-dose levofloxacin in combination with vancomycin is well tolerated and appeared to be efficacious in a patient with refractory multiresistant pneumococcal pericarditis. Vancomycin plus cephalexin [1, 4–7] is also current standard therapy of skin and skin-structure infections (CSSIs) caused by gram-positive bacteria. Chloramphenicol combining with vancomycin against vancomycin-resistant enterococci (VRE) [8] is also considered.

 Monitoring of vancomycin and cephalexin concentration in human plasma is essential for obtaining sufficient efficacy as well as preventing side effects. Various chromatographic methods have been developed for the determination of vancomycin individual in plasma samples [2]. Those include HPLC with UV detection [9–14], LC/MS [15–17], LC with electrochemical detection [18], and micellar electrokinetic capillary chromatography [19]. Furuta et al. [9] reported the direct injection method of plasma sample using a semipermeable surface packing material column. Cass et al. [16] reported an automated determination method by LC/MS coupled with on line extraction. However, these methods are relatively complicated and inconvenient, and the peak of vancomycin in the chromatogram is often not clearly separated from plasma blank peaks. In this case, quantification with univariate calibration will become biased and inaccurate. At the same time, simultaneous quantitative analysis of vancomycin and its combination therapy drug will make the analysis more difficult. However, by analyzing the HPLC-DAD data of unresolved peaks incorporating chemometric tools, it is possible to resolve those questions [20].

Ideally, each chromatographic peak corresponds to a single compound for HPLC analysis in optimized separation conditions. Generally, the retention factor (*k*) of the different analytes is between 1 and 20 allowing their complete separation. A peak with *k* equal to 0 is a component that does not interact with the stationary phase and elutes in the void volume. Chromatographic separations can become a difficult task when the samples are environmental and biological ones which have a complex matrix effects. The chromatographic peak of analyte maybe overlaps with the interferences or the other analytes. Particularly, the chromatographic resolution generally becomes poorer and partially separated peaks often occur when the speed of chromatographic analyses is increased, for example, by using higher flows, shorter columns, and so forth. On the other hand, it will be time-consuming and material-costing when completely chromatographic separation is carried out for the complex samples. At this time, applying mathematical separation as a complementary of chromatographic separation [21, 22] for resolving overlapping peaks is very promising. In chemometrics, three-way data analysis method can be used to resolve this question [21]. For HPLC-DAD, DAD can record the UV-Vis spectra at every retention time, and a matrix (elution time × wavelength) is obtained for every sample analyzed. A cube matrix can be obtained by detecting multiple samples simultaneously. The spectra of the calibration samples are measured at the different retention times of the peak of the analyte of interest and the retention times are selected by analyzing either a pure standard or a sample with a known added concentration of the analyte. The spectra of the predicted samples are measured at the different retention times of the peak from the unknown sample. Then the concentration of the analyte of interest in the overlapping peaks can be quantified by applying second-order calibration algorithms to the cube matrix.

**Figure 1:**
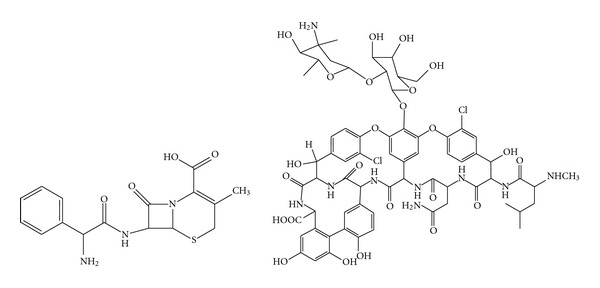
The structures of vancomycin and cephalexin.

In this paper, simultaneous and direct quantitative analysis of vancomycin and cephalexin in human plasma samples by using HPLC-DAD coupled with second-order calibration algorithms was developed.  Figure 1 lists the structures of the Vancomycin and cephalexin. The calibration results obtained with PARAFAC-ALS and self-weight alternative trilinear decomposition (SWATLD) were compared. These methods allow quantifying directly vancomycin and cephalexin concentration in complex human sample matrices without being influenced by the interference from the components. In the following part, bold capital letter for matrices, bold lowercase for vectors, and italics for scalars were used.

## 2. Theory

### 2.1. Trilinear Model for Second-Order Resolution

The mathematical formulation of HPLC-DAD for *N* components samples can also be expressed as follows:

(1) $$\begin{array}{c} x_{ijk}=\overset{N}{\underset{n=1}{\sum }}a_{in}b_{jn}c_{kn}+e_{ijk}\\ \left(i=1,\ldots ,I;\;j=1,\ldots ,J;\;k=1,\ldots ,K\right), \end{array}$$

where *x*_*ijk*_ is the intensity of the *k*th sample at *i*th chromatographic retention time and at *j*th wavelength. *N* is the total number of detectable components. *a*_*in*_, *b*_*jn*_, and *c*_*kn*_ are the elements of the loading matrices **A**, **B**, and **C**, respectively. **A** and **B** are the spectra and chromatographic profiles matrices for all *N* components, respectively. **C** denotes the relative concentration matrix. These can be expressed as **A** = (**a**_1_, **a**_2_,…, **a**_**n**_),  **B** = (**b**_1_, **b**_2_,…, **b**_*n*_), and **C** = (**c**_1_, **c**_2_,…, **c**_*n*_). *e*_*ijk*_ is the element of the measurement error matrix which contains the variation not captured by the model.

In contrast with bilinearity, the previous equation can be considered to be trilinearity. It can be viewed as an extension of Beer's law to second-order data [23]. This amounts to assuming that the measured peak is the sum of the individual peaks of each analyte and that the profile and the spectrum of one analyte are proportional in all the samples. As a consequence, the decomposition of a three-way data array built with response matrices measured for a number of samples is often unique, allowing chromatographic profile and spectral profiles, as well as relative concentrations of individual sample components to be extracted directly. Numbers of three-way methods used to resolve multicomponent mixtures have been proposed. They include the generalized rank annihilation method (GRAM) [24], direct trilinear decomposition (DTLD) [25], parallel factor analysis (PARAFAC) [26], alternating trilinear decomposition (ATLD) [27], coupled vectors resolution (COVER) [28], and self-weighted alternating trilinear decomposition (SWATLD) [29], multivariate curve resolution coupled to alternating least squares (MCR-ALSs) [30]. Due to the possibility of making analytical determinations even in the presence of nonmodelled interferents (this is known as the second-order advantage) [31] and to identify the analyte of interest, three-way algorithm is becoming increasingly important in routine analysis. In the second-order calibration algorithms that allow quantification even in the presence of noncalibrated components, the PARAFAC-ALS and SWATLD methods [32–34] are proved to be very useful for chromatographic data.

### 2.2. PARAFAC-ALS

PARAFAC is a generalization of principal component analysis (PCA) to higher orders. In PARAFAC, each component is trilinear, in contrast with bilinear PCA, where one score and one loading vector are obtained for each component. Three loading vectors (**a**_*n*_, **b**_*n*_, **c**_*n*_) are therefore given for each PARAFAC component. The PARAFAC model of a three-way array is found by minimizing the sum of the squares of the residuals *e*_*ijk*_. Each PARAFAC component gives three loadings: one relates to the chromatographic profile (**a**_*n*_), one relates to the spectral profile (**b**_*n*_), and one relates to the content of the samples (**c**_*n*_). Hence one loading is given for each dimension in the data. The algorithm used to solve the PARAFAC model is alternating least squares [26]. ALS successively assumes the loadings in two modes and then estimates the unknown set of parameters of the last mode. The algorithm converges iteratively until the relative change in fitting between two iterations is below a certain value (the default is 10^−6^). It is initialized by either random values or values calculated by a direct trilinear decomposition based on the generalized eigenvalue problem. Constraining the PARAFAC solution can sometimes be helpful in terms of the interpretability or the stability of the model. The resolution of spectra used to require the nonnegativity constraint since negative spectral parameters do not make sense.

### 2.3. SWATLD

 The PARAFAC algorithm is based on a least-square minimization, whereas SWATLD uses a procedure known as alternating trilinear decomposition [29]. The underlying theories have been recently reviewed [35]. Comparing with the PARAFAC algorithm, the SWATLD algorithm has the advantages of fast convergence and insensitivity to the excess factors used in calculations [23]. According to some experience, it offers better results than other second-order algorithms. There is a more detailed explanation of the algorithm in [29, 35].

All computer programs were written in the MATLAB (MathWorks) programming environment, and all calculations were carried out on a personal computer (Pentium IV processor).

## 3. Experimental

### 3.1. Reagents and Solutions

Vancomycin was purchased from Sigma (St Louis, MO, USA). Cephalexin was purchased from National Institute for the Control of Pharmaceutical and Biological Products in Changsha. Ethyl acetate was purchased from Sinochem Group ShangHai Corporation. Experiment water is doubly distilled water. All other reagents were of analytical grade. The stock solution was prepared by dissolving the vancomycin and cephalexin in the doubly distilled water, respectively. They are all stored in glass at 4°C.

### 3.2. Preparation of Human Plasma Samples

Human plasma samples received from the center of blood in changsha obtained from the healthy people were used to prepare predicted samples. Six of 2 mL samples of plasma were combined with various concentrations of the vancomycin and cephalexin stock solutions and ethyl acetate were added. Thereafter, the mixture was shaken in a vortex for 30 s and centrifuged at 3500 rpm for 5 min, and the organic phase was evaporated, respectively. Then, doubly distilled water was added to these flasks in order to obtain 10 mL solution.  Table 1 displays the concentration of six synthetical plasma samples. The plasma fraction was collected and stored at 4°C until analyzed.

### 3.3. HPLC Instrumentation and Conditions

Throughout the analysis was carried out using an HPLC system (Agilent Series 1100, Agilent Technologies, Palo Alto, CA, USA), which consists of vacuum degasser, autosampler, and a binary pump, equipped with a Zorbax Eclipse XDB-C8 analytical column (125 × 4.0 mm; 5 um). Theoretically, the analytes of interest can be determined precisely by changing the experimental conditions to achieve full resolution. This involves spending time and resources, and there is no guarantee that the separation will be complete. According to our experiments, the completely separation of vancomycin and cephalexin is difficult to carry out. It is also noticed that the chromatographic peak shape of vancomycin will become wide when we try to separate vancomycin and cephalexin by adjusting the mobile phase. This will make the last calibration result inaccuracy. Therefore in our experiment, the following chromatographic conditions are applied. Column temperature was maintained at 30°C. Mobile phases A and B were methanol and 0.1 M disodium hydrogen phosphate buffer (40/60 v/v %), respectively. The flow-rate used was kept at 1.0 mL/min in each study. The wavelength used was 200.0 nm to 380.0 nm with an interval of 2.0 nm. The run time used was in the range of 0 min~5 min (1/30 min intervals). The baseline effect is compensated by subtracting the measurement matrix of an average blank from the sample measurement.

Sixteen samples are divided into two sets; that is, the first ten samples are the calibrated sets (no human plasma) and the remaining six are the predicted set. The human plasma was only added to six predicted samples (drug-free plasma). After eliminating the noninformation data obtained with HPLC-DAD, three-way data arrays (250 × 50 ×10) were treated by using the PARAFAC-ALS and SWATLD, respectively.

## 4. Results and Discussion

### 4.1. Number of Factors

The chemical rank is defined as the number of significant factors distinguishing from noise. When using PARAFAC, an initial definition of the number of factors to build the model is necessary. This choice is of fundamental importance because all conclusions about the deconvolution and quantitation results will be related with this number of factors. The analytes in the mixture are often unknown in practical analysis process. Therefore, deciding the underlying species in the mixture is always the key step to further qualitative and quantitative analysis. Some methods have been suggested to estimate the chemical rank of the three-way data arrays. Bro and Kiers [36] suggested obtaining the number of responsive components (*N*) by consideration of the internal parameter known as core consistency diagnosis (CORCONDIA), which is a measure of how well a given model is able to reproduce the so-called Tucker core of a cube of data. The core consistency is calculated as a function of a trial number of components. It remains near a value of 100 when the number is less than or equal to the optimum; for exceeding component numbers it drops below 50%.

**Figure 2:**
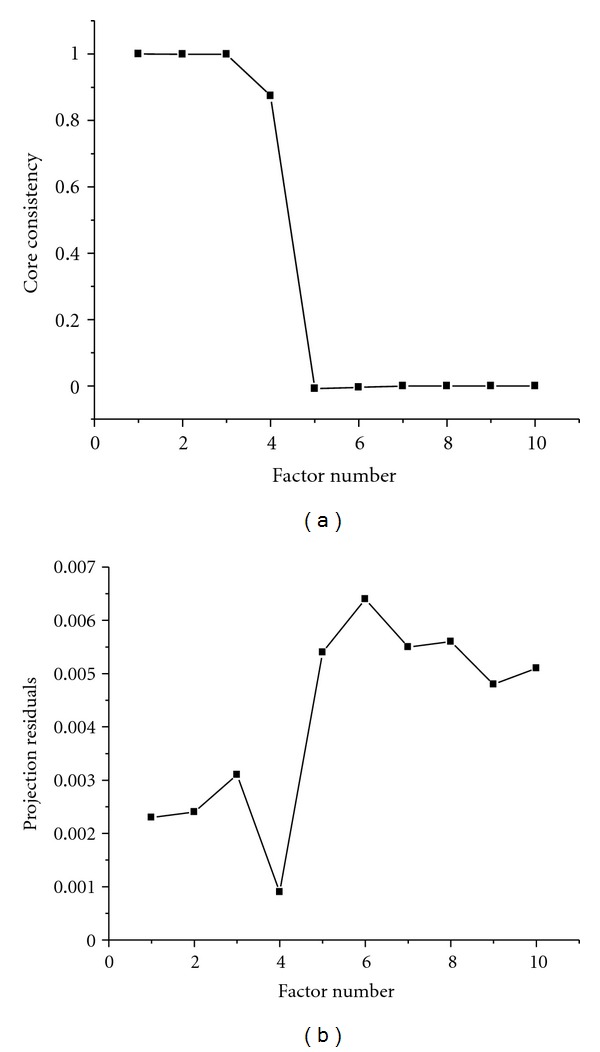
The results of factor-determining of the HPLC-DAD data arrays by CORCONDIA (a) and LTMC (b).

Unconstrained PARAFAC-ALS models of this HPLC-DAD data were developed using one to ten components, and the percentage of fit is used as the initial approach to select the number of factors.  Figure 2(a) shows the value obtained for the 16-sample cube when studying the human plasma sample. As can be seen, the core consistency drops to a very low value when using five components to model the cube, suggesting that *N* = 4 is a sensible choice. It shows that the human plasma introduces some interferences to the mixture system.

On the other hand, anyone of these methods cannot ensure to obtain the accurate result for a practical mixture system. Two or more methods are often used to estimate the appropriate component number of the mixture to confirm the result [19]. We also suggest a simple linear transform incorporating Monte Carlo simulation approach (which names LTMC) to determine the component number of the three-way data arrays [37]. The newly proposed method decides the chemical rank through performing a simple linear transform procedure to the original cube matrix to produce two subspaces by singular value decomposition: one of two subspaces is derived from the original three-way data array itself and the other is derived from a new three-way data array produced by the linear transformation of the original one. Projection technique incorporating the Monte Carlo approach acts as distinguishing criterion to chose the appropriate component of the mixture. This method is also used in this experiment. Figure 2(b) also shows the result calculated by the LTMC. The projection residuals for the former four factors are relatively small but a rapid increase for the later factors. Because the first four factors represent the real factors spaces, the later factors represent the noise spaces. It indicates that the trilinear data arrays request to be fitted exactly with four factors. This is coincident with the result obtained by CORCONDIA.

### 4.2. PARAFAC-ALS and SWATLD Analysis

**Figure 3:**
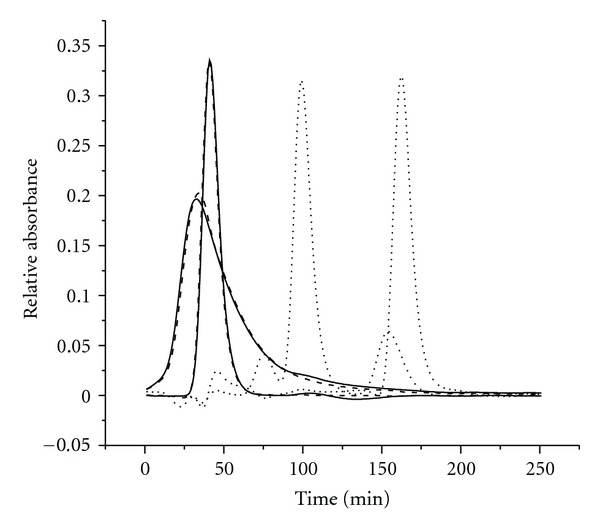
Normalized chromatographic profiles were resolved by the SWATLD in human plasma. Solid and dotted lines represent the chromatographic profiles of vancomycin, cephalexin, and interference. The medium dash denotes the actual vancomycin and cephalexin.

**Figure 4:**
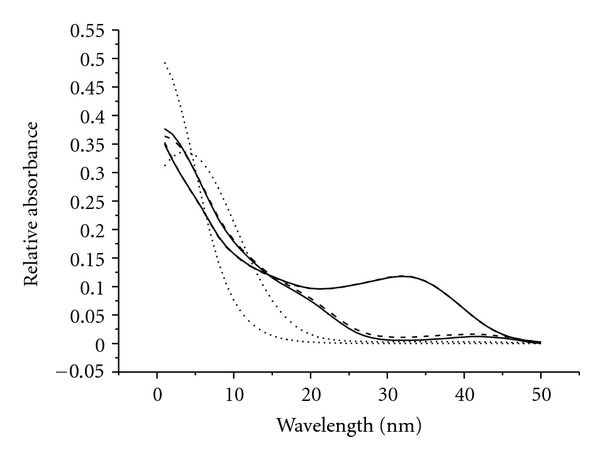
Normalized UV-Vis spectral profiles were resolved by the SWATLD in human plasma. Solid and dotted lines represent the UV-vis spectral profiles of vancomycin, cephalexin, and interference. The medium dash denotes the actual vancomycin and cephalexin.

Once the appropriate component number is correctly determined, three-way HPLC-DAD data for the sixteen samples were analyzed by PARAFAC-ALS and SWATLD, respectively. Specific details on the implementation of PARAFAC and SWATLD methods will be given as follows. Figures 3 and 4 show the chromatographic profile and UV-Vis spectra profile obtained with SWATLD. The chromatography and spectra of pure vancomycin and cephalexin were obtained by measuring the pure vancomycin and cephalexin and decomposing them by singular value decomposition (SVD). It is also shown in Figures 3 and 4. We regarded these normalized profiles as the spectra of the pure and used them as reference spectra to evaluate the reliability of the models in the calibration. The correlation coefficients which calculate the pure spectrum and the ones resolved by SWATLD all exceed 0.998 for the each individual analyte in this paper. It shows that the SWATLD is reliable to resolve the HPLC-DAD data. The result of the PARAFAC-ALS is not given for its sameness with SWATLD.

Two means can be used to decide on the concentration of the predicted samples (unknown sample). Type one adopts the following:

(2) $$\hat{c}=\mathrm{diag}\left(A^{+}X_{un}{\left(B^{T}\right)}^{+}\right).$$

Here **A** and **B** denote the chromatographic profile and spectral profile, and **X**_*un*_ denotes the data matrix which comes from an unknown sample. It is noticeable that the interference included in the unknown samples should be contained in matrices A and B. Otherwise the result will be inaccurate. This type is often used in BLLS and GRAM. In PARAFAC and SWATLD, type two is often applied; that is, the calibration step and predicted step are synchronous implemented. The relative concentration *c*_*Kn*_ can be acquired by running two algorithms with the other loading. The unknown concentration ${\hat{y}}_{un}$ of the *n*th component can be obtained by regressing it based on the known standard concentration *y* of the *n*th component:

(3) $$\begin{array}{c} k=y^{+}\times \left[c_{1n}\;|\;c_{2n}\;|\;\cdots \;|\;c_{Kn}\right],\\ {\hat{y}}_{un}=\frac{c_{un}}{k}. \end{array}$$

Here “+” denotes the pseudoinverse. *c*_*Kn*_ denotes the concentration of the *n*th component in the *K*th sample.

 The prediction results for the human plasma samples are listed in Table 1. The accuracy of the models was calculated by the root mean square error of prediction (RMSEP):

(4) $$\text{RMSEP}=\sqrt{\frac{\sum_{i=1}^{m}{\left(y_{i}-{\hat{y}}_{i}\right)}^{2}}{m}},$$

where *y*_*i*_ and ${\hat{y}}_{i}$ are the added and predicted concentrations of the given vancomycin or cephalexin in the *i*th prediction sample, and *m* is the number of prediction samples. RMSEP was 0.81% for vancomycin and 0.95% for cephalexin with SWATLD and 0.89% for vancomycin and 1.02% for cephalexin with PARAFAC-ALS. It can be found that the root mean square error of prediction of two algorithms is very coincident. The SWATLD is only a slight superior to the PARAFAC-ALS.

These values can also be expressed in terms of recovery (as the percentage ratio between the predicted and the true concentration) so that they could be compared with two algorithms (Table 1). The average recoveries for the PARAFAC-ALS procedure are 101 ± 5% for vancomycin and 96 ± 3% for cephalexin. For SWATLD, the average recoveries are 102 ± 4% for vancomycin and 97 ± 3% for cephalexin, respectively. So, in all the cases, recoveries are around the ideal 100% for both methods; it shows that the dispersion of the results was lower for SWATLD than that for PARAFAC-ALS. On the same time, the iterative numbers of SWATLD are shorter than PARAFAC-ALS (SWATLD only requests 15 times but 478 times for PARAFAC-ALS) and insensitive for the chemical rank of the mixture system. This is coincident with the former application.

### 4.3. Figures of Merit

Alternative methodology needs to be validated by comparison with the other established methods. The most important process for comparison of analytical methods is the determination of figures of merit (FOM), such as sensitivity, selectivity, and limit of detection (LOD). In multivariate calibration, the net analyte signal (NAS) calculation [31] is strictly necessary for the FOM evaluation. The NAS for multiway data is analogous to those for first-order procedures, which is defined as the part of the signal that relates uniquely to the analyte of interest. In this case, as the data are bilinear, the NAS is the pure analyte data obtained by PARAFAC [38]. The sensitivity is estimated as the NAS at unit concentration, as shown in (5), and the selectivity is the ratio between the sensitivity and the total signal, as shown in (6):

(5) $$\begin{array}{c} \text{SEN}=k{\left\{{\left[{\left(A^{T}A\right)}^{-1}\right]}_{nn}{\left[{\left(B^{T}B\right)}^{-1}\right]}_{nn}\right\}}^{-1/2}, \end{array}$$

(6) $$\begin{array}{c} \text{SEL}={\left\{{\left[{\left(A^{T}A\right)}^{-1}\right]}_{nn}{\left[{\left(B^{T}B\right)}^{-1}\right]}_{nn}\right\}}^{-1/2}, \end{array}$$

where** A** and **B** are the matrices which collect spectral and chromatographic profiles for all *N* components, respectively. *n* denotes the *n*th component in mixture system. *k* denotes the regression coefficient calculated as aforementioned. The limit of detection (LOD) [38] is calculated as

(7) $$\text{LOD}={3.3s}_{\left(\text{o}\right)},$$

where *s*_(o)_ is the standard deviation from the concentration estimated for three different blank samples in the PARAFAC and SWATLD models, respectively. The more details can be found [38]. In second-order calibration, different analytes are not separated completely by chromatography as tradition performance. Thus the linear range has not been studied in this paper. The figures of merit of vancomycin and cephalexin for PARAFAC-ALS and SWATLD are displayed in Table 2. As can be observed, the figures of merit for both PARAFAC-ALS and SWATLD are very similar and only the sensitivity of SWATLD is greater than that of PARAFAC-ALS. In terms of the figures of merit, cephalexin is more selective than vancomycin as its spectra are the most different in shape (see Figures 2 and 3) from the others and therefore the least correlated. This preliminary information suggests that cephalexin will be predicted at lower concentrations (it is the more sensitive). This is confirmed with the LOD calculated.

## 5. Conclusion

Simultaneous and direct determination of vancomycin and cephalexin in human plasma by coupling HPLC-DAD with second-order calibration algorithms was developed in this paper. This approach allows quantifying vancomycin and cephalexin under the incompletely chromatographic separation and presenting unknown inference in the prediction samples. Slightly better results in the plasma samples analysis are obtained by application of SWATLD calibration compared to PARAFAC-ALS. The satisfactory recoveries were obtained in all cases when several plasma samples were analyzed. This work demonstrated that the use of HPLC-DAD coupled with second-order calibration algorithms is a powerful tool to quantify overlapped chromatographic profiles for complex analysis of drugs in plasma. At the same time, the methodology involving PARAFAC and SWATLD did not require as many calibration samples as the PLS models do and, what is more, would allow the determination of any of the vancomycin and cephalexin in the presence of unknown interferences (second-order advantage) even if they were not included in the model. Extremely important issues such as reduction in the time of analysis and consequently costs and amount of contaminant solvents should also be considered. The figures of merit calculated for both PARAFAC and SWATLD were very similar and the results should be considered satisfactory based on the complexity of the samples analyzed. They are acceptable for some real applications, such as pharmacokinetic investigations in patients.

## Figures and Tables

**Table 1:** Determination results of vancomycin and Cephalexin by HPLC-DAD using PARAFAC-ALS and SWATLD algorithm in human plasma (*N* = 4).

Sample	Added		SWATLD		PARAFAC	
	Van^c^	Cep^d^	Van^c^	Cep^d^	Van^c^	Cep^d^
	(mg/mL)	(mg/mL)	(mg/mL)	(mg/mL)	(mg/mL)	(mg/mL)
11	15.15	15.27	14.25	14.71	14.02	14.57
12	20.20	20.36	20.58	20.52	20.06	20.49
13	30.30	10.18	30.64	9.99	30.39	10.02
14	20.20	30.54	21.52	29.46	21.50	29.27
15	25.25	25.45	25.24	23.68	24.81	23.71
16	20.20	15.27	21.25	14.39	21.45	14.25

11			94.1%	96.4%	92.5%	95.4%
12			101.9%	100.8%	99.3%	100.7%
13			101.1%	98.1%	100.3%	98.5%
14			106.6%	96.5%	106.5%	95.8%
15			99.9%	93.0%	98.2%	93.2%
16			105.2%	94.2%	106.2%	93.3%
MR^a^			102 ± 4%	97 ± 3%	101 ± 5%	96 ± 3%
RMSEP^b^			0.0081	0.0095	0.0089	0.0102

^a^Mean recovery.

^b^Root mean square error of prediction.

^c^Vancomycin.

^d^Cephalexin.

**Table 2:** Analytical figure of merit.

	SWALTD		PARAFAC	
Figure of merit^a^	Vancomycin	Cephalexin	Vancomycin	Cephalexin
Sensitivity (SEN), ABS, mL/mg	1.009	1.920	0.8921	1.301
Selectivity (SEL)	0.105	0.235	0.102	0.238
LOD, mg/mL	0.32	0.047	0.12	0. 026

^a^ABS is the absorption intensity (arbitrary units).
